# Primary care incidence and treatment of four neuropathic pain conditions: A descriptive study, 2002–2005

**DOI:** 10.1186/1471-2296-9-26

**Published:** 2008-05-06

**Authors:** Gillian C Hall, Dawn Carroll, Henry J McQuay

**Affiliations:** 1 Grimsdyke House, London, UK; 2 Outcomes Research and Evidence Based Medicine, Pfizer Ltd, Tadworth, UK; 3 University of Oxford Pain Relief Unit, The Churchill, Oxford, UK

## Abstract

**Background:**

Between 1992 and 2001 the UK general practice incidence of post-herpetic neuralgia and trigeminal neuralgia declined, whilst the incidence of painful diabetic neuropathy increased. The most common first line treatments were compound analgesics. As therapeutic options have subsequently changed, this study presents updated data on incidence and prescribing patterns in neuropathic pain.

**Methods:**

A descriptive analysis of the epidemiology and prescription treatment at diagnosis of incident post-herpetic neuralgia (n = 1,923); trigeminal neuralgia (1,862); phantom limb pain (57) and painful diabetic neuropathy (1,444) using computerised UK general practice records (THIN): May 2002 to July 2005.

**Results:**

Primary care incidences per 100,000 person years observation of 28 (95% confidence interval (CI) 27–30) for post-herpetic neuralgia, 27 (95%CI 26–29) for trigeminal neuralgia, 0.8 (95%CI 0.6–1.1) for phantom limb pain and 21 (95%CI 20–22) for painful diabetic neuropathy are reported. The most common initial treatments were tricyclic antidepressants (post-herpetic neuralgia) or antiepileptics (trigeminal neuralgia and painful diabetic neuropathy) and opioid analgesics (phantom limb pain). The mean number of changes before a stable drug regimen was 1.2 to 1.5 for trigeminal neuralgia, painful diabetic neuropathy and post-herpetic neuralgia, and 2.4 for phantom limb pain.

**Conclusion:**

The incidence of phantom limb pain and post-herpetic neuralgia are decreasing whilst painful diabetic neuropathy plateaued and trigeminal neuralgia remained constant. Despite more frequent use of antidepressants and antiepileptics for first line treatment, as opposed to conventional non-opioid analgesics, changes to therapy are common before a stable regimen is reached.

## Background

A fifth of adults in Europe have moderate or severe chronic pain but, although only 2% are managed by a specialist [1], most published data on the epidemiology and treatment of pain conditions come from secondary care sources [2-6]. The prevalence of pain of predominantly neuropathic origin has been reported as 8% in a UK primary care survey [7]. One UK General Practice Research Database study investigated the epidemiology and treatment of four neuropathic pain syndromes; post-herpetic neuralgia, trigeminal neuralgia, phantom limb pain and painful diabetic neuropathy [8]. Data from 1992 to 2002 showed trends in incidence of disease, with painful diabetic neuropathy and trigeminal neuralgia increasing over the ten years while post-herpetic neuralgia and phantom limb pain decreased. Co-proxamol, carbamazepine, amitriptyline, codeine plus paracetamol and co-dydramol were the five most common medications included in initial treatments across all four conditions. Within the more commonly prescribed therapies, those patients who received an antiepileptic or an antidepressant at the time of diagnosis were shown to be less likely to switch therapy.

It was anticipated that prescribing patterns for the neuropathic pain conditions would have changed recently with negative publicity concerning co-proxamol [9], increased use of newer therapies such as gabapentin, and changes to prescribing guidance [10]. Continuing changes in incidence rates may have an impact on workload. The objective of this study was to investigate current prescribing patterns and to update the incidence estimates for neuropathic pain syndromes.

## Methods

The study was an observational, descriptive study of patients with an incident diagnosis of one of four neuropathic pain syndromes. All data were obtained from the Health Information Network database (THIN), which is an observational database containing primary care records from throughout the UK. Details of demographics, primary care diagnoses and prescription treatment are routinely recorded against date in individual patient records. Information on referrals, secondary care diagnoses and deaths are also captured because of the structure of the UK National Health Service (NHS). Within the NHS, the general population are registered with one General Practitioner (GP) and remain on that GP's list whilst being treated by hospital specialists or hospitalized. Major events from before computerization are added retrospectively. Medical events are recorded using the Read Coding system [11].

### Study Populations

The study population comprised all patients who were permanently registered at one of the practices contributing to THIN at any time in the study period, May 2002 to July 2005; that is over 2.9 million patients from 295 practices, 51% female and 20% over 60 years of age. The files of all patients in the study population were searched for a record of any of the four forms of neuropathic pain studied [see Additional file 1]. The index date was the date of the first record of the neuropathic pain or neuropathy. Patients could be in more than one category. A post-herpetic neuralgia record was a specific term for post-herpetic neuralgia or an acute herpes zoster term plus either neuropathy, or neuropathic pain, 3–6 months after the first acute herpes zoster entry. A trigeminal neuralgia record had a specific term for this diagnosis. Phantom limb pain was defined as a specific term or a term for amputation plus either a neuropathy or neuropathic pain record 3–24 months after the first amputation code. Patients were included in the painful diabetic neuropathy cohort if their record contained a specific term; a term for diabetic neuropathy with a prescription for a treatment for pain current at the date of diagnosis; a record of diabetes and a general term for neuropathic pain or record of diabetes and both neuralgia and a treatment for pain current on the date of the neuralgia code. A neuropathic pain treatment was defined as an analgesic (excluding low dose aspirin), an anaesthetic (oral or intravenous), an antiepileptic with no history of epilepsy, or an antidepressant. A sensitivity analysis was included in the study of painful diabetic neuropathy to assess the impact of removing patients with a prescription for a treatment for pain, current at the date of diagnosis, but not initiated on this date. This group may have included patients with non-painful diabetic neuropathy who were being treated for depression or other painful conditions.

The person years observation and incidence per person years observation were estimated for each form of neuropathic pain. Those with a first record of their neuropathic pain dated during their period of observation counted as incident cases and formed the neuropathic pain cohorts. A period of observation was assigned for each patient. The start of observation was the later of May 2002, or one year after the date that either the practice started using the Vision Computer System or the patient registered at that practice. The year was added to allow time for the recording of prevalent events. The end of observation was the earliest of the following: death, transfer-out of the practice, the final data collection or the end of July 2005. Incidence rates were age adjusted by direct standardisation to the UK figures for 2005 [12].

### Treatment Patterns

The computerised treatment records of each patient in the incident neuropathic pain cohorts were searched for an initial treatment, defined as any treatment for neuropathic pain started within 28 days of the first record of the diagnosis. If more than one therapy was prescribed in this period then that nearest to the first record was the initial treatment. When more than one item was prescribed on the same day, the initial treatment was considered to be the combination of these therapies. For the four study conditions, the number of patients with each initial drug regimen (drug and daily dose), therapeutic class, and number of therapies per patient were counted. Therapeutic class was based on the British National Formulary [13]. Daily doses which could not be interpreted from the computer record were grouped, as were those that were interpreted but were not specific (for example: '2–3, 3–4 times a day', 'as directed' or 'as required'). The latter group was labelled 'dose not specified'. When a prescription described a dose titration, then the final dose was included. The duration was calculated for each initial treatment. A therapy was considered to have stopped when no additional prescription was issued within 56 days, a concomitant drug was introduced or, for combination therapies, one therapy was stopped. When the duration could not be calculated from details on a prescription, it was assumed to last 28 days.

To understand changes in treatment a sub-group of patients who received an initial treatment and who could be followed for at least one further year were identified. Prescription records during the year were searched for any changes in neuropathic pain treatment. Changes to therapy included each switch, new treatment, discontinuation or addition of a concomitant drug. A switch was defined as prescriptions for more than one drug, where the prescription for the first drug was dated before that of the second, with less than 56 days between these prescriptions, and no subsequent treatment for the first drug in the 56 days after the prescription for the second drug. A new treatment was defined as prescriptions for more than one drug, where one prescription was dated after the other, with between 56 days and 6 months between the prescriptions and no subsequent treatment for the first drug in the 56 days after the second prescription. Concomitant therapy was defined as prescriptions for more than one treatment where the date of the second prescription was on or after the starting treatment date but on or before the date of the last prescription in a treatment episode (one or more prescriptions separated by less than 56 days). A discontinuation was defined as no further prescriptions for that drug. Stable therapy was defined as more than two prescriptions for the same drug or drugs (ignoring daily dose) with less than 56 days between them, and without a break of more than 6 months with no neuropathic pain treatment. The number of patients who reached a stable therapy regimen within the year was counted.

Ethics approval was given by London MREC, reference number 05/MRE02/77.

## Results

Incident cases of one or more of the four neuropathic pain conditions were identified for 5,445 patients during the period of observation. There were 1,923 incident cases of post-herpetic neuralgia, incidence 28.2 per 100,000 person years (95% CI 27.0, 29.5) and 1,862 cases of trigeminal neuralgia, incidence 27.3 (95% CI 26.1–28.6) and 57 of phantom limb pain, incidence 0.8 (95% CI 0.6–1.1). The incidence of painful diabetic neuropathy was 27.2 (95% CI 26.0–28.4), n = 1,867, and 26.1 per 100,000 person years when the stricter definition was used excluding those whose pain treatment did not start at the time of the first diagnosis record. The age standardised incidence rates (to UK 2005) were 27.3 per 100,000 person years for post herpetic neuralgia, 26.7 for trigeminal neuralgia, 0.8 for phantom limb pain and 26.7 for painful diabetic neuropathy.

A treatment for neuropathic pain was initiated at the time of the first diagnosis record for between 49.4% and 73.5% of patients with a neuropathic pain condition (Table 1). The mean duration of this treatment varied between 50 days for post-herpetic neuralgia and 90 days for trigeminal neuralgia. The tricyclic antidepressant amitriptyline was the most frequently prescribed treatment for post-herpetic neuralgia, phantom limb pain and painful diabetic neuropathy, and the second most commonly prescribed in trigeminal neuralgia. Carbamazepine an antiepileptic was the most common item on the initial prescription for those diagnosed with trigeminal neuralgia (Table 1). The majority of patients were prescribed one treatment when therapy was initiated so the majority of patients received one therapeutic class of drug (Table 2). At the time of the first record, 46.8% of patients with trigeminal neuralgia started treatment with carbamazepine, however 52.7% were being treated with this drug. Antidepressants and antiepileptics were prescribed in an initial treatment to the majority of patients: 75% of patients with trigeminal neuralgia, 63% with painful diabetic neuropathy or phantom limb pain, and 57% of those with post-herpetic neuralgia (Table 3). Between 55 and 68% of the treated cohorts had at least one year of data after this initial treatment. For those who reached stable therapy during this period the median number of preceding changes was two for trigeminal neuralgia and painful diabetic neuropathy and three for post-herpetic neuralgia and phantom limb pain (Table 3). There was great variation in initial treatments with no treatment – dose combination given to more than nine percent of patients (Table 4).

**Table 1:** Initial treatments^a ^by pain condition

	Post Herpetic Neuralgia	Trigeminal Neuralgia	Phantom Limb Pain	Painful Diabetic Neuropathy
Number with an initial treatment (% total with condition)	1,414 (73.5)	1,164 (62.5)	40 (70.2)	922 (49.4)
Duration of initial treatment^b ^in days mean (± SD)	50.5 (126.3)	90.0 (168.6)	73.1 (192.9)	77.1 (141.2)

**Items prescribed on initial prescription as % of those treated**				
1 item	56.2	72.6	52.5	63.7
2 items	24.6	15.9	40.0	24.2
3 or more items	19.2	11.5	7.5	12.1

**Five most common medications included in initial treatments, n (% treated)**				
1	Amitriptyline hydrochloride 704 (49.8)	Carbamazepine 545 (46.8)	Amitriptyline hydrochloride 12 (30.0)	Amitriptyline hydrochloride 375 (40.7)
2	Codeine phosphate +paracetamol 248 (17.5)	Amitriptyline hydrochloride 304 (26.1)	Gabapentin 9 (22.5)	Gabapentin 154 (16.7)
3	Capsaicin 149 (10.5)	Codeine phosphate +paracetamol 131 (11.3)	Paracetamol 6 (15.0)	Codeine phosphate+ paracetamol 112 (12.1)
4	Co-dydramol 126 (8.9)	Gabapentin 99 (8.5)	Codeine phosphate+ paracetamol 5 (12.5)	Paracetamol 97 (10.5)
5	Carbamazepine 118 (8.3)	Co-proxamol 59 (5.1)	Carbamazepine 3 (7.5)	Co-dydramol 54 (5.9)

^a^Started within 28 days of the first record of the disease. ^b^Duration of initial treatment in days, ignoring titration of dose. SD, standard deviations

**Table 2:** Initial treatments by condition and combination of therapeutic class (percentage of treated patients)

	**Post-herpetic neuralgia**	**Trigeminal neuralgia**	**Phantom limb pain**	**Painful diabetic neuropathy**
	N (%)	N (%)	N (%)	N (%)
Opioid analgesics alone	84 (5.9)	45 (3.9)	7 (17.5)	47 (5.1)
Opioid analgesics + Antidepressants (tricyclic)	41 (2.9)	12 (1.0)	2 (5.0)	21 (2.3)
Opioid analgesics + Antidepressants (other)	2 (0.1)	1 (0.1)	0	4 (0.4)
Opioid analgesics + Antiepileptics	13 (0.9)	22 (1.9)	0	8 (0.9)
Opioid analgesics + Non-opioid analgesics	168 (11.9)	94 (8.1)	5 (12.5)	97 (10.5)
Opioid analgesics + Rubefacients	6 (0.4)	1 (0.1)	0	2 (0.2)
Antidepressants (tricyclic) alone	445 (31.5)	225 (19.3)	8 (20.0)	281 (30.5)
Antidepressants (tricyclic) + Antidepressants (other)	2 (0.1)	1 (0.1)	0	5 (0.5)
Antidepressants (tricyclic) + Antiepileptics	7 (0.5)	12 (1.0)	0	14 (1.5)
Antidepressants (tricyclic) + Non-opioid analgesics	68 (4.8)	21 (1.8)	1 (2.5)	35 (3.8)
Antidepressants (tricyclic) + Rubefacients	17 (1.2)	2 (0.2)	0	2 (0.2)
Antidepressants (tricyclic) + Local anaesthetics	2 (0.1)	0	0	0
Antidepressants (other) alone	7 (0.5)	7 (0.6)	2 (5.0)	23 (2.5)
Antidepressants (other) + Antiepileptics	2 (0.1)	10 (0.9)	1 (2.5)	8 (0.9)
Antidepressants (other) + Non-opioid analgesics	4 (0.3)	1 (0.1)	1 (2.5)	5 (0.5)
Antidepressants (other) + Rubefacients	3 (0.2)	0	0	0
Antiepileptics alone	158 (11.2)	543 (46.7)	7 (17.5)	165 (17.9)
Antiepileptics + Non-opioid analgesics	15 (1.1)	13 (1.1)	3 (7.5)	5 (0.5)
Antiepileptics + Rubefacients	8 (0.6)	2 (0.2)	0	5 (0.5)
Antiepileptics + Local anaesthetics	2 (0.1)	1 (0.1)	0	0
Non-opioid analgesics alone	70 (5.0)	43 (3.7)	1 (2.5)	72 (7.8)
Non-opioid analgesics + Rubefacients	7 (0.5)	0	0	2 (0.2)
Rubefacients alone	78 (5.5)	12 (1.0)	0	37 (4.0)
Local anaesthetics alone	2 (0.1)	4 (0.3)	0	8 (0.9)
1 therapeutic category	844 (59.7)	879 (75.5)	25 (62.5)	633 (68.7)
2 therapeutic categories	367 (26.0)	193 (16.6)	13 (32.5)	213 (23.1)
3 or more therapeutic categories	203 (14.4)	92 (7.9)	2 (5.0)	76 (8.2)

**Table 3:** Number of changes^a ^from initial^b ^to stable^c ^therapy by condition and therapeutic class included in the prescription

	**Number of patients**			**Changes to stable therapy in 1 year**	
		
**Initial Treatment Includes:**	**Treated (% all treated^d^)**	**Followed >1 year (% treated)**	**Stable in 1 year (%followed)**	**Median**	**Range**
**Post-herpetic neuralgia:**					
**Total**	1414	828 (58.6)	232 (28.0)	3	0–20
Antiepileptics	248 (17.5)	153 (61.7)	46 (30.1)	3	1–20
Antidepressants (tricyclics)	728 (51.5)	422 (58.0)	104 (24.6)	2	0–11
Antidepressants (other)	39 (2.8)	26 (66.7)	10 (38.5)	4	2–8
Non-opioid analgesics	529 (37.4)	310 (58.6)	103 (33.2)	3	0–18
Opioid analgesics	504 (35.6)	277 (55.0)	92 (33.2)	3	0–18
Rubefacients/other topical antirheumatics	150 (10.6)	91 (60.7)	29 (31.9)	2	1–8
Local anaesthetics	8 (0.6)	4 (50.0)	2 (50.0)	3	1–4

**Trigeminal neuralgia:**					
**Total**	1164	762 (65.5)	168 (22.0)	2	0–17
Antiepileptics	653 (56.1)	433 (66.3)	91 (21.0)	2	0–17
Antidepressants (tricyclics)	318 (27.3)	205 (64.5)	43 (21.0)	2	1–12
Antidepressants (other)	29 (2.5)	16 (55.2)	4 (25.0)	2	1–5
Non-opioid analgesics	258 (22.2)	169 (65.5)	40 (23.7)	2	0–17
Opioid analgesics	260 (22.3)	165 (63.5)	45 (27.3)	3	1–17
Rubefacients/other topical antirheumatics	24 (2.1)	14 (58.3)	4 (28.6)	6	2–12
Local anaesthetics	5 (0.4)	3 (60.0)	1 (33.3)	4	4-4

**Phantom limb pain:**					
**Total**	40	27 (67.5)	13 (48.1)	3	1–53
Antiepileptics	11 (27.5)	8 (72.7)	2 (25.0)	3	2–3
Antidepressants (tricyclics)	13 (32.5)	8 (61.5)	5 (62.5)	3	1–36
Antidepressants (other)	4 (10.0)	3 (75.0)	3 (100.0)	1	1–53
Non-opioid analgesics	13 (32.5)	8 (61.5)	3 (37.5)	1	1–3
Opioid analgesics	16 (40.0)	9 (56.3)	5 (55.6)	3	1–5
Rubefacients/other topical antirheumatics	0	-	-	-	-
Local anaesthetics	0	-	-	-	-

**Painful diabetic neuropathy:**					
**Total**	922	508 (55.1)	161 (31.7)	2	0–31
Antiepileptics	237 (25.7)	107 (45.1)	41 (38.3)	2	0–16
Antidepressants (tricyclics)	404 (43.8)	233 (57.7)	73 (31.3)	2	0–12
Antidepressants (other)	59 (6.4)	30 (50.8)	12 (40.0)	4	1–8
Non-opioid analgesics	283 (30.7)	171 (60.4)	48 (28.1)	2	0–31
Opioid analgesics	249 (27.0)	137 (55.0)	54 (39.4)	2	0–31
Rubefacients/other topical antirheumatics	54 (5.9)	34 (63.0)	14 (41.2)	2	1–8
Local anaesthetics	9 (1.0)	8 (88.9)	3 (37.5)	1	1–7

^a^Treatment changes comprised each switch, discontinuation, new treatment or addition of a concomitant treatment, but not a change in dose. ^b^Started within 28 days of the first record of the disease. ^c^More than two prescriptions for the same treatment with less than 56 days between them. ^d ^Some patients had more than 1 therapeutic class in the initial therapy, percentages add to >100.

**Table 4:** Number of changes^a ^from initial^b ^to stable^c ^therapy by condition for initial treatments (drug(s) and dose) prescribed to ≥ 10 patients

**Initial treatment**	**Patients**			**Changes to stable therapy**		
		
**Drug (dose)**	**Treated (% all treated)**	**Followed >1 year (% treated)**	**Stable in 1 year (% followed)**	**Mean**	**Median**	**Range**
**Post-herpetic neuralgia**						
Amitriptyline hydrochloride (10 mg per day)	93 (6.6)	50 (53.8)	9 (18.0)	2.6	2	1–5
Amitriptyline hydrochloride (10 mg not specified)	65 (4.6)	36 (55.4)	4 (11.1)	4.8	5	2–8
Amitriptyline hydrochloride (25 mg per day)	64 (4.5)	39 (60.9)	10 (25.6)	2.6	3	1–5
Codeine phosphate (8 mg)+paracetamol (500 mg not specified)	34 (2.4)	21 (61.8)	9 (42.9)	4	2	1–12
Co-dydramol (not specified) Dihydrocodeine tartrate (10 mg)+paracetamol (500 mg)	30 (2.1)	20 (66.7)	7 (35.0)	5.6	4	1–18
Codeine phosphate (30 mg)+paracetamol (500 mg not specified)	24 (1.7)	15 (62.5)	5 (33.3)	5.4	5	1–9
Capsaicin (0.025% 4 times a day)	19 (1.3)	15 (78.9)	6 (40.0)	2.8	2	1–6
Carbamazepine (200 mg per day)	19 (1.3)	12 (63.2)	7 (58.3)	4	4	1–8
Paracetamol (500 mg not specified)	18 (1.3)	14 (77.8)	4 (28.6)	2.8	2	1–6
Co-proxamol (not specified) Dextropropoxyphene hydrochloride (32.5 mg)+paracetamol (325 mg)	17 (1.2)	11 (64.7)	2 (18.2)	5	5	3–7

**Trigeminal neuralgia**						
Carbamazepine (200 mg per day)	103 (8.8)	67 (65.0)	9 (13.4)	3.7	3	1–10
Amitriptyline hydrochloride (10 mg per day)	53 (4.6)	35 (66.0)	6 (17.1)	2	2	1–4
Amitriptyline hydrochloride (10 mg not specified)	43 (3.7)	33 (76.7)	3 (9.1)	1.3	1	1–2
Carbamazepine (300 mg per day)	35 (3.0)	22 (62.9)	8 (36.4)	2.1	2	1–4
Carbamazepine (100 mg not specified)	33 (2.8)	19 (57.6)	4 (21.1)	1.5	2	1–2
Amitriptyline hydrochloride (25 mg per day)	31 (2.7)	17 (54.8)	4 (23.5)	3.5	3	2–6
Carbamazepine (100 mg per day)	30 (2.6)	22 (73.3)	4 (18.2)	2.5	2	1–5
Carbamazepine (400 mg per day)	29 (2.5)	23 (79.3)	5 (21.7)	4.8	2	1–16
Gabapentin (300 mg per day)	21 (1.8)	14 (66.7)	5 (35.7)	2.8	3	1–4
Codeine phosphate (8 mg)+paracetamol (500 mg not specified)	20 (1.7)	12 (60.0)	3 (25.0)	5.7	6	5–6
Codeine phosphate (30 mg)+paracetamol (500 mg not specified)	19 (1.6)	13 (68.4)	4 (30.8)	2	1	1–5
Co-proxamol (not specified) Dextropropoxyphene hydrochloride (32.5 mg)+paracetamol (325 mg)	14 (1.2)	11 (78.6)	1 (9.1)	1	1	1-1

**Painful diabetic neuropathy**						
Amitriptyline hydrochloride (10 mg per day)	72 (7.8)	35 (48.6)	4 (11.4)	1.5	2	1–2
Amitriptyline hydrochloride (25 mg per day)	41 (4.4)	25 (61.0)	7 (28.0)	3	2	1–11
Amitriptyline hydrochloride (10 mg not specified)	35 (3.8)	21 (60.0)	2 (9.5)	1	1	1-1
Gabapentin (900 mg per day)	30 (3.3)	15 (50.0)	5 (33.3)	5.2	4	2–12
Co-dydramol (not specified) Dihydrocodeine tartrate (10 mg)+paracetamol (500 mg)	21 (2.3)	15 (71.4)	4 (26.7)	2.8	1	1–8
Paracetamol (500 mg not specified)	20 (2.2)	11 (55.0)	1 (9.1)	1	1	1-1
Codeine phosphate (8 mg)+paracetamol (500 mg not specified)	18 (2.0)	13 (72.2)	1 (7.7)	2	2	2-2

^a^Treatment changes comprised each switch, discontinuation, new treatment or addition of a concomitant treatment, but not a change in dose. ^b^Started within 28 days of the first record of the disease. ^c^More than two prescriptions for the same treatment with less than 56 days between each one.

Within those patients in the phantom limb pain cohort, and followed for at least one year, no initial treatment (drug and daily dose combination) had been prescribed to ten or more patients. For post-herpetic neuralgia and painful diabetic neuropathy the most common drug and dose combinations were amitriptyline 10 or 25 milligrams per day and 10 milligrams 'dose not specified'. Carbamazepine 200 milligrams per day was the most common treatment for trigeminal neuralgia, followed by amitriptyline 10 milligrams per day and 10 milligrams 'dose not specified'.

## Discussion

This large study aimed to provide updated incidence rates and prescribing practices for four neuropathic pain conditions as seen by UK general practitioners rather than in secondary care clinics. The population was similar to that of the UK in terms of age and sex, 51% were female as in the UK 2001 census data, while there were slightly fewer elderly patients, 20% over 60 years of age compared to 21% [14].

The incidence rate of 28.2 per 100,000 patient years for post-herpetic neuralgia is in-line with other published findings [15,16] which give rates of 34 and 49 per 100,000 person years one month after acute herpes zoster. When compared to data from a similar general practice study, the incidence had decreased since 1998–2001, age-adjusted incidence of 56.8 per 100,000 population compared to 27.3 in 2002–2005 [8]. This may relate to the definition of PHN which included a GP diagnosis of post-herpetic neuralgia. A GP's perception of post herpetic neuralgia may include acute or sub-acute zoster pain. In addition to inflating the true incidence rate, if perceptions change with time and possibly education, this could explain the declining rates. Alternatively, treatment of acute herpes zoster with antiviral agents has been reported to reduce both the risk of developing post-herpetic neuralgia and the overall the duration of pain [17]. No UK data were available on the use of antivirals in herpes zoster, but information from the Netherlands suggest that, while they are used in a minority of patients (22.5%), treatment is more common in those at higher risk of complications [18]. Without intervention the rising age of the UK population might be expected to result in higher rates of post-herpetic neuralgia as age is a risk factor for both acute herpes zoster and post-herpetic neuralgia in acute herpes zoster [19].

Our finding of an incidence of trigeminal neuralgia of 27 per 100,000 person years is consistent with the previous database study, however these rates are considerably higher than those previously published (2.1 to 4.7 per 100,000 patient years and 8 per persons per annum [20-22]). This further supports the view that primary care physicians use a wide definition when they diagnose trigeminal neuralgia. The incidence of phantom limb pain of 0.8 per 100,000 person years is slightly less than the 1998–2001 rate of 0.9 [8] (age-adjusted rates of 0.8 per 100,000 person years and 1.3 in 1998–2001) as might be expected given the lower rate of limb amputations [23]. No other community based incidence rates were found for phantom limb pain.

Incidence rates for painful diabetic neuropathy, from both this and the previous UK general practice study are considered to be maximum incidences. The data source did not allow separation of patients with non-painful diabetic neuropathy who had a treatment for depression or other pain initiated at the same time as the neuropathy was first recorded from those treated for painful diabetic neuropathy. Although the crude incidence of painful diabetic neuropathy increased, age-adjusted rates (taking into account the new dataset) were the same for the 1998–2001 and 2002–2005 data.

### Neuropathic pain treatment

A treatment was initiated at the time of diagnosis for between 40% and 74% of patients in the neuropathic cohorts. Other patients may have been established on a treatment before a firm diagnosis was made, particularly as definitions for both phantom limb pain and post-herpetic neuralgia include a delay between the precipitating event and diagnosis. The prescribing of antidepressants and antiepileptics is in-line with UK guidelines for neuropathic pain treatment [13,24], although amitriptyline is not included in these recommendations for the first line treatment of trigeminal neuralgia. Amitriptyline is the tricyclic antidepressant of choice in all cohorts despite evidence that imipramine and nortriptyline are also effective [24]. Prescribing paracetamol alone, or in combination with codeine, is recommended for post-herpetic neuralgia patients with mild to moderate pain [10]. Our finding that combination therapy was more common than paracetamol alone could, in part, be due to the availability of over the counter paracetamol.

When compared to prescribing in the previous, similar UK primary care study [8] the 2002 to 2005 data show an increase in the use of antidepressants and antiepileptics while prescribing of conventional non-opioid analgesics declined. Across all four conditions there was a two to four fold increase in use in amitriptyline between the January 1992 to April 2002 and May 2002 to July 2005 cohorts (1992-April 2002 data:12%, 6%, 8% and 24% for post-herpetic neuralgia, trigeminal neuralgia, phantom limb pain and painful diabetic neuropathy respectively). Gabapentin is now one of the five most common initial treatments in three of the conditions, the exception being post-herpetic neuralgia, and has replaced carbamazepine as the most commonly prescribed antiepileptic for phantom limb pain and painful diabetic neuropathy. Gabapentin was not licensed for neuropathic pain until 2000 and so was not a frequently prescribed treatment for any condition in the earlier analysis. Carbamazepine use increased only in the treatment of post-herpetic neuralgia. While the negative press reports concerning the association of co-proxamol and fatal overdose may have accounted for some of the change, the decreased use of all non-opioid analgesics is probably due to increased evidence and acceptance of efficacy of other agents [25-29]. The withdrawal of co-proxamol was not announced until February 2005 so will have had little effect on this study. Another change was the inclusion of the rubefacient capsaicin in the top five most frequently prescribed items for post-herpetic neuralgia, possibly because it was not licensed for neuropathic pain for all of the earlier study period and was not included in previous management recommendations.

The study used the most up to date records available. This allowed us to provide current incidence figures, but meant that we had one year of follow-up on 56% to 66% of the cohorts. The remainder will either have left the practice before the end of the year or have been diagnosed within a year of either the last data collection or the end of the study period. Fewer patients with post-herpetic neuralgia and trigeminal neuralgia were followed to stable regimen than those with phantom limb pain and painful diabetic neuropathy. This difference may be partly the result of our definition rather than an indication that patients with post-herpetic neuralgia and trigeminal neuralgia have more difficulty achieving effective pain relief. Stable therapy was defined as more than two prescriptions for the same treatment. As post-herpetic neuralgia may wane while trigeminal neuralgia can be intermittent these patients may not require a third consecutive prescription.

Results of the January 1992 to April 2002 analysis suggested that, for the most commonly prescribed treatments, changes to a treatment regimen appeared to be less frequent when initial therapy was with frequently used antidepressants or antiepileptics rather than compound analgesics. Our ability to explore this further in more recent cohorts is limited given that conventional analgesics were prescribed less frequently and severity of pain or underlying disease cannot be accounted for in the dataset. However, the mean change in therapy within cohorts was slightly greater in this study (2–3 changes compared to 1–2 changes) despite a larger proportion receiving antiepileptics and antidepressants. Conversely, the mean duration of the initial treatment increased, 50 to 90 days compared to 47 to 76 days previously, as did the number of initial prescriptions with more than one item (28% to 43% compared to 7% to 19% previously) [8].

## Conclusion

Incidence rates of phantom limb pain and post-herpetic neuralgia as seen in UK general practice continue to change, although the reasons for this are not clear. Treatment patterns at the time of the first diagnosis have also changed as the use of antidepressant and antiepileptics has increased and is common across all conditions studied with most patients prescribed one item. Although this is in line with directives, frequent changes to therapy before a stable regimen is reached suggest that pain relief with tolerability are difficult to achieve.

## Abbreviations

THIN: The Health Information Network database; NHS: National Health Service; GP: General Practitioner; UK: United Kingdom.

## Competing interests

Gillian Hall has received funding for research and payment for consultancy from a number of pharmaceutical companies and charities and has no direct stock holding in any pharmaceutical company. Dawn Carroll was an employee of Pfizer Inc. Henry McQuay has received research support, consultancy and lecture fees from pharmaceutical companies, charities and government sources, and has no direct stock holding in any pharmaceutical company.

## Authors' contributions

All authors formulated the research questions. GH participated in the design of the study and was responsible for the analysis and interpretation of the patient database and drafting and finalising the paper. DC and HMcQ participated in the design of the study and commented on the paper. All authors read and approved the final manuscript.

## Pre-publication history

The pre-publication history for this paper can be accessed here:



## Supplementary Material

Additional file 1 Read codes with terms used for identification of four neuropathic pain cohorts. Click here for file
